# *Trypanosoma evansi* infection in Tunisia: current situation

**DOI:** 10.1051/parasite/2025058

**Published:** 2025-10-06

**Authors:** Mohamed Gharbi, Meha Kamoun, Médiha Khamassi, Syrine Rekik, Boubaker Ben Smida, Jawhar Fekih Ahmed, Chayma Boubaker, Giuliano Cecchi, Geoffrey Gimonneau, Marc Desquesnes

**Affiliations:** 1 IDEALISS ULR 7519, École vétérinaire UniLaSalle de Rouen 76130 Mont-Saint-Aignan France; 2 Laboratory of Parasitology, University Manouba, National School of Veterinary Medicine of Sidi Thabet 2020 Sidi Thabet Tunisia; 3 Laboratory of Infectious Animal Diseases, Zoonoses and Sanitary Regulation, University Manouba, National School of Veterinary Medicine of Sidi Thabet 2020 Sidi Thabet Tunisia; 4 Arrondissement de la Production Animale de Tataouine 3263 Tataouine Tunisia; 5 Arrondissement de la Production Animale de Mahdia 5111 Mahdia Tunisia; 6 Animal Production and Health Division (NSA), Food and Agriculture Organization of the United Nations (FAO) Rome Italy; 7 Institut Sénégalais de Recherches Agricoles, Laboratoire National de l’Élevage et de Recherches Vétérinaires route du Front de Terre Dakar Hann Sénégal; 8 CIRAD, UMR INTERTRYP 34398 Montpellier France; 9 INTERTRYP, Université de Montpellier, CIRAD, IRD Montpellier France; 10 CIRAD, ENVT 31300 Toulouse France

**Keywords:** *Trypanosoma evansi*, Epidemiology, Control, Vector, One Health, Tunisia

## Abstract

Surra is a vector-borne disease, caused by a flagellate protozoan, *Trypanosoma evansi*, infecting all domestic mammals, including herbivores and dogs, and, very rarely, humans. In Tunisia, it affects mainly dromedaries (*Camelus dromedarius*) in the southern part of the country, causing heavy economic losses due to high morbidity, abortions and mortality. *Trypanosoma evansi* is mainly transmitted by mechanical vectors (Stomoxyine flies and tabanids), but also vertically, orally (to carnivores) and iatrogenically. In the present paper, we review and discuss the studies published on surra in Tunisia and show that the antibody seroprevalence in Tunisian dromedaries varies between 22.2% and 37%. The review also highlights the absence of a comprehensive database containing the most relevant information on the occurrence of *T. evansi* in Tunisia. We also underscore the urgent need for data collection and analyses. These data should be related to different aspects: epidemiological data (spatial and temporal distribution) and entomological data (main vectors involved in the transmission and their activity dynamics).

## Abstract (fr)

Le surra est une maladie vectorielle, causée par un protozoaire flagellé, *Trypanosoma evansi*, qui infecte tous les mammifères domestiques, y compris les herbivores et les chiens, et, très rarement, l’homme. En Tunisie, il touche principalement les dromadaires (*Camelus dromedarius*) dans le sud du pays, entraînant de lourdes pertes économiques dues à une morbidité, des avortements et une mortalité élevés. *Trypanosoma evansi* est principalement transmis par des vecteurs mécaniques (Stomoxes et Tabanidae), mais aussi verticalement, oralement (aux carnivores) et par voie iatrogène. Cet article passe en revue et discute les études publiées sur le surra en Tunisie et montre que la séroprévalence des anticorps chez les dromadaires tunisiens varie entre 22,2 % et 37 %. L’étude souligne également l’absence d’une base de données exhaustive contenant les informations les plus pertinentes sur la présence de *T. evansi* en Tunisie, et souligne le besoin urgent de collecte et d’analyse de données. Ces données devraient être liées à différents aspects : données épidémiologiques (distribution spatiale et temporelle) et données entomologiques (principaux vecteurs impliqués dans la transmission et leur dynamique d’activité).

## Introduction

**Figure 1:**
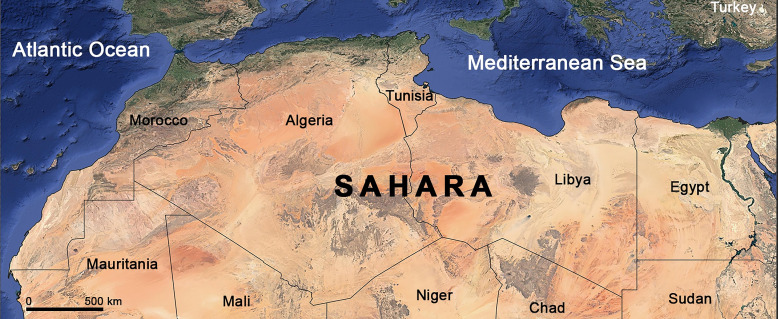
Map of North Africa showing the geographic location of the Sahara.

Tunisia is a North African country of 163,610 km^2^ sharing borders with Algeria (1,010 km) and Libya (459 km) (Fig. 1). To date, the only pathogenic *Trypanosoma* reported in Tunisia is *T. evansi*, the causative agent of surra [5]. North Africa is out of the geographic distribution of tsetse flies (*Glossina* spp.), sometimes referred to as “the tsetse belt” [3]. These flies are the cyclical vectors of animal and human African trypanosomosis (AAT and HAT, respectively). AAT, or nagana, is normally considered to include infections with *Trypanosoma vivax*, *Trypanosoma congolense*, *Trypanosoma brucei* and *Trypanosoma simiae* [10, 42]. Among these, *T. vivax* is the only species that managed to spread very far from the tsetse belt in sub-Saharan Africa, like in south and central America, and more recently in Iran [1], thanks to its ability to be transmitted mechanically by other hematophagous flies [13]. To date, there is no information about the presence of *T. vivax* in Tunisia. On the other hand, *Theileria lestoquardi*, a sub-Saharan piroplasm that affects small ruminants, was reported in Tunisia [30], demonstrating the presence of pathogen flow between North Africa and sub-Saharan countries. This flow is associated with persistent ancestral movement of humans and domestic animals, mainly dromedaries, across the whole Sahara which is tolerated and even encouraged by several countries.

*Trypanosoma evansi* is transmitted by blood sucking flies such as Tabanidae, Stomoxinae and blood-sucking *Musca* spp. flies, such as *Musca crassirostris* [6]. Even though surra is a notifiable disease to the World Organisation of Animal Health (WOAH) and has been known for several decades by locals as *Debad,* the Arabic name of “fly”, scanty information is available on this disease in Tunisia.

**Figure 2:**
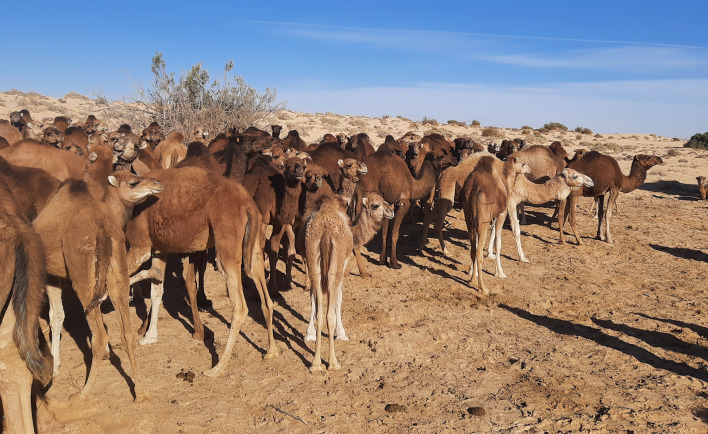
Dromedaries in Tataouine, South Tunisia around a drinking source. Note the presence of small “islands” of humidity and organic matter from animal faeces, representing a suitable microhabitat for the development of larvae of different blood-sucking flies.

The Tunisian Sahara constitutes, according to the adopted classification, between 33% and 40% of the total area of Tunisia [27]. In the Tunisian Sahara, dromedaries are maintained under two main types of husbandry systems: peri-urban, where animals are kept in small areas around households in villages and suburbs, and pastoral, where animal graze in the Sahara for most of the year and are grouped only for breeding [9]. In some regions, animals are periodically grouped around water sources for drinking and for mating (Fig. 2). Dromedaries play a very important socio-economic role since they represent the main, and sometimes the only source of income. They may be the sole food source (milk and meat), and provide wool, leather, labour, and transportation. In several coastal areas, they are used in tourism for riding. A high infection prevalence of *T. evansi* clearly affects, especially when causing chronic disease, all these resources and incomes. This worsens the socio-economic situation of inhabitants of the Sahara since they represent a vulnerable population that lives with limited food resources in extreme climate conditions with very large temperature ranges, extreme aridity and sandy winds that can last for several days [35]. Treatment of infected animals is poor, especially in the southern regions, because it is administered late. This occurs since most dromedary owners live far from veterinary care services.

## Epidemiology of surra in Tunisia

In Tunisia, surra mainly affects dromedaries (*Camelus dromedarius*) that are concentrated in the Southern part of the country, belonging to the Sahara. In addition, there are periodically documented clinical cases in other parts of the country. Although surra is considered to be present in all Mediterranean African countries, including Tunisia, [17], there were no official reports of its presence in Tunisia before the 1990s.

There are unexpected surra clinical cases in Tunisia and others could be cryptic, especially in sheep and cattle. Some sheep and dromedary owners from the Sahara migrate northward during summer to rent wheat straw lands.

Dromedaries are exposed to the vector’s bites on a seasonal basis, because, in the Sahara, the abiotic conditions are favourable to the insects only during a specific period of the year. Even though the seasonality of clinical cases has not been scientifically documented, it is known that there are two incidence peaks, one in summer and the second in autumn (Regional veterinary services of Tataouine, Unpublished data).

**Figure 3:**
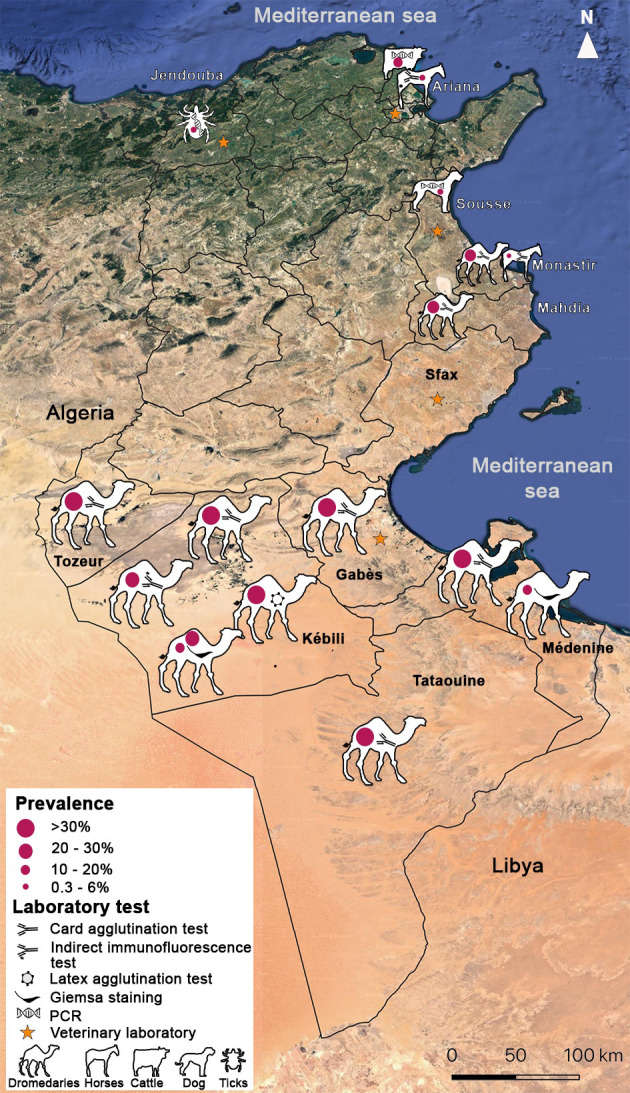
Map of Tunisia showing the geographic distribution of different cases of *Trypanosoma evansi* infection in different governorates. The prevalence for each species and the technique used are indicated for each governorate.

Epidemiological surveys on *T. evansi* infection in Tunisia have been sporadic, using different screening techniques. There is no systematic declaration of clinical cases (Fig. 3). A study carried out in 1992, investigating the one-year seasonality of seropositivity among dromedaries in Kebili district (south-west), showed that the highest values of seroprevalence were recorded in spring (March) 45.97% (40/87) and autumn (November) 32.4% (35/108), respectively by indirect immunofluorescence (IFI) and card agglutination test for trypanosomes: CATT/*T. evansi* [2].

Another study in the same region of Kebili district, conducted in 1997 among a semi-intensive dromedary herd belonging to the tourism ministry, revealed a seroprevalence of 42.6% (128/300) and 36.3% (32/88) by Suratex^®^ (latex agglutination test) and CATT/*T. evansi* [4], respectively. Thirty-one blood samples (10.33 ± 1.75; 31/300) showed positivity on direct examination of Giemsa-stained blood smears (also seropositive on the latex agglutination test) and the animals displayed variable symptoms such as anaemia, enlarged lymph nodes and emaciation [4]. Seroprevalence in Tunisian dromedaries varies between regions, and depends on the serological test used. Based on CATT/*T. evansi*, seroprevalence in dromedaries ranges from 0 to 42.66 ± 2.85% (128/300) in Tataouine [4] (Table 1).

**Table 1:** Exhaustive list of studies estimating the prevalence of *Trypanosoma evansi* infection in Tunisia.

Governorate	Animal species	Technique	Prevalence in % (positive/examined)	Reference
Ariana	Cattle	Polymerase chain reaction	10 ± 0.03 (10/96)	Sallemi *et al.*, 2017 [36]
Gabès	Dromedaries (*Camelus dromedarius*)	Agglutination test for trypanosomiasis	22.2 ± 0.6 (4/18)	Sana *et al.*, 2022 [37]
	Dromedaries (*Camelus dromedarius*)	Agglutination test for trypanosomiasis	28.6 ± 0.3 (14/49)	Sana *et al.*, 2022 [37]
Jendouba (National Park of El Feidja)	Ticks in environment	Polymerase chain reaction	0.3 ± 0.1 (1/279)	Said *et al.*, 2021 [34]
	*Rhipicephalus sanguineus* sensu lato (males)	Polymerase chain reaction	5.8 ± 0.4 (1/18)	Said *et al.*, 2021[34]
Kebili	Dromedaries (*Camelus dromedarius*)	– Agglutination test for trypanosomiasis	19.62 ± 2.05 (73/372)	Azzabi, 1993 [2]
		– Indirect immunofluorescence test	24.46 ± 2.22 (91/372)	
	Dromedaries (*Camelus dromedarius*)	– Giemsa-stained blood smears	10.33 ± 1.75 (31/300)	Cherfeddine, 1998 [4]
		– Agglutination test for trypanosomiasis	36.66 ± 5.12 (32/88)	
		– Latex agglutination test (Suratex)	42.66 ± 2.85 (128/300)	
	Dromedaries (*Camelus dromedarius*)	Giemsa-stained blood smear	22% ± 0.4 (22/100)	Said and Yahia, 2019 [32]
	Dromedaries (*Camelus dromedarius*)	– Giemsa-stained blood smears	5.4 ± 2.1% (4/47)	Ismail-Hamdi *et al.*, 2022 [18]
		– Agglutination test for trypanosomiasis		
	Dromedaries (*Camelus dromedarius*)	– Giemsa-stained blood smears	5.4 ± 2.1% (4/47)	Ismail-Hamdi *et al.*, 2022 [18]
		– Agglutination test for trypanosomiasis		
	Dromedaries (*Camelus dromedarius*)	– Agglutination test for trypanosomiasis	37.5 ± 0.3 (144/384)	Sana *et al.*, 2022 [37]
Medenine	Dromedaries (*Camelus dromedarius*)	– Giemsa-stained blood smears	4.5 ± 1.1 (4/97)	Ismail-Hamdi *et al.*, 2022 [18]
		– Agglutination test for trypanosomiasis		
	Dromedaries (*Camelus dromedarius*)	– Giemsa-stained blood smears	3.9 ± 0.2 (4/103)	Ismail-Hamdi *et al.*, 2022 [18]
		– Agglutination test for trypanosomiasis		
	Dromedaries (*Camelus dromedarius*)	Agglutination test for trypanosomiasis	26.2 ± 0.3 (86/328)	Sana *et al.*, 2022 [37]
Southern Tunisia*	Dromedaries (*Camelus dromedarius*)	– Indirect immunofluorescence test	20.6 ± 0.4 (26/126)	Elandalousi *et al.*, 2013 [11]
Sousse	Dog	– Giemsa-stained blood smears	1 dog	Rjeibi *et al.*, 2015 [31]
		– Polymerase chain reaction		
Tataouine	Dromedaries (*Camelus dromedarius*)	– Agglutination test for trypanosomiasis	25.4 ± 0.3 (80/315)	Sana *et al.*, 2022 [37]
	Dromedaries (*Camelus dromedarius*)	– Giemsa-stained blood smears	0 (0/50)	Ismail-Hamdi *et al.*, 2022 [18]
		– Agglutination test for trypanosomiasis		
	Dromedaries (*Camelus dromedarius*)	– Giemsa-stained blood smears	0	Ismail-Hamdi *et al.*, 2022 [18]
		– Agglutination test for trypanosomiasis		
Tozeur	Dromedaries (*Camelus dromedarius*)	Agglutination test for trypanosomiasis	36 ± 0.4 (40/111)	Sana *et al.*, 2022 [37]
Northern (Ariana, Bizerte, Jendouba, Béja, Kef) and Central (Monastir, Mahdia) Tunisia	Equids (horses, donkeys and mules)	Agglutination test for trypanosomiasis	2.3 ± 1.6 (2/87)	Joober, 2024 [21]

* Governorate was not indicated in the paper.

In 2012, an outbreak of surra was reported in hotel riding dromedaries in Sahline (governorate of Monastir). Four dromedaries out of 22 died and 6 females aborted. Different symptoms of surra were reported in all dromedaries, including weight loss, anaemia (haematocrit reached 18%), depilation, diarrhoea, adenomegaly, epiphora and a pseudo ebrious gait [15]. All sera were strongly positive to CATT/*T. evansi*. The same year, 14 hotel riding dromedaries in Mahdia governorate manifested clinical signs of surra, and all were positive for CATT/*T. evansi* (Fekih Ahmed, Personnel observation). In El Jem (governorate of Mahdia), the regional veterinary services performed screening for 25 healthy dromedaries belonging to a dromedary trader, 8 were positive to CATT/*T. evansi* (Fekih Ahmed, Personnel observation). In the same governorate, on a farm holding 19 dromedaries, 8 animals developed symptoms of surra: hyperoxia, diarrhoea, wool loss and 5 abortions (Fekih Ahmed, Personnel observation).

Later on, an epidemiological survey was performed in cattle, near a touristic region in Ariana, North Tunisia, where hotels also use dromedaries for riding. The molecular prevalence in tested cattle was 10% (10/96), and as far as it could be ascertained, no clinical surra cases were reported in this locality [36].

The study by Lachtar *et al.* [25] showed that out of 254 dromedaries from 13 water gathering stations, 27 were positive (10.6%) using only microscopic examination of Giemsa-stained blood smears. In this study, the highest prevalence was recorded in February.

A recent study carried out on equids (mules, horses and donkeys) showed that out of 87 animals, two were positive (2.29 ± 1.6%) to CATT/*T. evansi*, both used for agriculture activities. The first seropositive case was a 10-year-old mare bred in a mixed flock with cattle, sheep, goats, dogs and chickens in the district of Ariana, North Tunisia. The second was a two-year-old mare and bred with cattle from Mahdia district (the coastal central-east region). These animals were asymptomatic [21].

In 2015, the first published Tunisian clinical surra case was in a dog living at a hotel located in Sousse region that became infected with *T. evansi* by ingesting the placenta of an aborted female dromedary used for tourist riding by the hotel. Initially, the clinical symptoms were confused with leishmaniosis (*Leishmania infantum* infection), which is endemic in Tunisia, but parasitological and molecular diagnoses confirmed *T. evansi* infection [31].

Except for these cases (dromedary, horse, dog and cattle), *T. evansi* has never been reported in other species including humans in Tunisia. Despite this, surra is considered to be a potential zoonotic disease, qualified as atypical human trypanosomosis (aHT) [24, 40], the number of documented human cases remains very low, compared to the relatively high seroprevalence in some regions of the world. Indeed, in India, 5.2% (9/173) of human blood samples were positive by CATT/*T. evansi* and 2.89% (5/173) were positive by PCR targeting the variable surface glycoprotein (VSG) gene sequences [39]. As far as could be ascertained, human cases of surra were never reported in Tunisia. However, at the same time and to the best of our knowledge, attempts at laboratory diagnostics have not been carried out in humans, although dromedary shepherds live continuously in the Sahara and, like their animals, are exposed to blood sucking flies. The social impact of surra on dromedary keepers in the Sahara is difficult to explore unless multidisciplinary teams are built including sociologists, veterinarians, medical doctors and animal breeders. Although several aspects of the social impact of surra, such as impact on human and animal welfare, depopulation of the Sahara, and the impact of the disease on the dromedary owner’s social prestige are difficult to estimate and quantify, they could be explored, analysed and discussed.

## Diagnosis of surra in Tunisia

Clinical signs in dromedaries are, in most cases, sufficient to conclude on *T. evansi* infection (surra). However, since symptoms are not pathognomonic, laboratory diagnosis is required to confirm suspicions. In Tunisia, there are six laboratories able to implement tests: one parasitology laboratory located at the National School of Veterinary Medicine of Sidi Thabet (*École Nationale de Médecine Vétérinaire de Sidi Thabet*) and five laboratories of the Institute of Veterinary Research of Tunisia (*Institut de Recherches Vétérinaires de Tunisie*) located in Tunis, Bou Salem, Sousse, Sfax and Gabès. The last is the nearest to the dromedary area and is located at approximately 100 km from the first southern town, Médenine. Due to the long distance between the animal keeping sites and the laboratories, delays occur in confirmation of diagnosis, making it more expensive to test. This often discourages animal owners and veterinarians from requesting laboratory analyses in the first place. In this situation, the veterinary practitioners most often proceed to therapeutic diagnosis, *i.e.* diagnosis *a posteriori* based on the effectiveness of administered drugs. Diagnosis in Tunisian laboratories relies on microscopic examination of Giemsa-stained blood smears, which is rapid, easy and inexpensive, but it is also of very poor sensitivity, *i.e.* only positive when parasitaemia exceeds 5 × 10^5^ parasites/mL [41]. Furthermore, this technique allows for detection of co-infections with several other haemoparasites (*Anaplasma* spp, *Theileria annulata, Theileria equi*, *Babesia caballi*, *Babesia vogeli, etc.*) and provides an excellent estimation of parasitaemia, helping the practitioner to take suitable decisions regarding the treatment to deliver. Other trypanosome species can also be found through Giemsa-stained blood smears (GSBS), especially *T. theileri* and *T. melophagium* in cattle and sheep. These are two non-pathogenic *Trypanosoma* of the sub-genus *Megatrypanum* that could be differentiated by their sizes from *T. evansi.* Of note, the size of *Megatrypanum* is more than 30 μm. However, these non-pathogenic parasites are hardly ever detected through GSBS microscopic examination because their parasitaemia is normally too low for parasites to be detected. It must be emphasized that more sensitive parasitological techniques such as the haematocrit centrifuge technique (HCT, also known as Woo technique) should be applied on a regular basis to increase the sensitivity of parasite detection. Here again, *Megatrypanum* can be distinguished from *T. evansi* based on their size and shape (very long and sharp posterior extremity) [43].

## Control of surra in Tunisia

In Tunisia, trypanosomosis in the dromedary is on the list of regulated animal diseases for which general policy measures must be applied when a case is confirmed (which rarely happens due to the lack of laboratory diagnosis), and notification in case of suspicion is mandatory [22]. However, mandatory notification is rarely applied since animals are often in remote areas.

The control of surra in Tunisia is limited to the treatment of clinical cases with melarsomine hydrochloride (Cymelarsan^®^). This trypanocidal drug is subsidized by the Government and is provided to farmers at 10% of its real price by regional Tunisian offices of livestock and pasture, Ministry of Agriculture (*Office de l’élevage et des pâturages*) (farmers pay only 6.500 TND corresponding to 1.96€ per dose). However, to obtain this treatment, farmers must present a positive laboratory analysis result, and face the constraints presented above. These conditions in the process of delivering trypanocides aim to limit trypanocide overuse and to prevent the development of *T. evansi* resistant strains, a problem also documented for other pathogenic trypanosomes and trypanocides in sub-Saharan Africa [12, 28, 42]. In 2024, a total of 200 doses of melarsomine hydrochloride were administered (*Arrondissement de la production animale de Tataouine*) which is probably far below the number of surra cases in Tunisia. This situation is due to the process of drug access that is long and costly, but also due to the availability of diminazene aceturate (DA) which is illegally imported from neighbouring countries at low prices (15 TND per dose corresponding to approximately 4,52€) and is available without restriction. Practically, the majority of Tunisian veterinary practitioners administer a trypanocide (either melarsomine or diminazene) with large spectrum antibiotics and vitamin C.

Spraying insecticides in the environment to control flies is not possible since dromedaries live in vast open spaces and a high number of hematophagous insect species with different phenology and behaviours may act as vectors. Insecticides would be certainly inefficient to eliminate these vectors and would have a very negative impact on non-targeted invertebrates and vertebrates. Importantly, the idea that the Sahara is an “empty” or “poor” ecosystem is totally false; there are several animal species, but their density is low and they are mostly hidden during the day [33, 34]. Acaricides are used on Tunisian dromedaries to control ticks and mange [20], but not to protect animals against biting insects. To be efficient, repeated and continuous applications of insecticides would be needed, making this control option tedious and expensive, with a high persistent negative impact on human, animal and environmental health. Use of insecticides is performed by animal owners without planned programmes, making these actions generally ineffective.

## Recommendations for better control of surra in Tunisia

### Diagnosis of *Trypanosoma evansi* infection

The haematocrit centrifugation technique (HCT) has higher sensitivity than microscopic examination of Giemsa-stained blood smears [8]. Unlike the GSBS technique, HCT is quick and cheap and its use would therefore be beneficial if it were generally applied by regional laboratories. To reduce the cost and the time of laboratory diagnosis, local laboratories with trained personnel could be established in different localities of South Tunisia. They could be provided with basic equipment.

Molecular techniques, particularly PCR, could be used to increase the sensitivity of agent detection tests, and to increase species specificity, notably to confirm whether only *T. evansi* is present, or if *T. vivax* (the agent of nagana which may also be mechanically transmitted by hematophagous flies) is also circulating. Serological tests such as CATT/*T. evansi* and ELISA *T. evansi* could be used for epidemiological studies even though they do not allow differentiation between past and current infection [7]. The seroprevalence of *T. evansi* infection in Southern Tunisia is high, so serological positive results may not be very informative in clinically infected animals, since serology does not make a distinction between infected and diseased animals. A parasitological test based on HCT and/or a molecular test based on the PCR would be recommended. However, serological techniques would be highly informative in investigating the northern area of the country, and especially other livestock species, especially ruminants, which may more easily behave like healthy carriers of *T. evansi*, regardless of the geographical area concerned.

### Epidemiological studies in Tunisia

As reported above, some information on the geographic distribution of surra clinical cases is available in Tunisia; however, its systematic documentation in a national atlas, as was developed for nagana in many African countries [14, 29] and more recently also for surra in Spain [26], would help decision-makers and practitioners to improve their knowledge about the epidemiology and distribution of surra, notably to evaluate the risk in relation to hosts and geographical area concerned.

It is of paramount importance to develop studies on *T. evansi* and possible *T. vivax* infections to estimate different epidemiological indicators, mainly seroprevalence and the incidence of clinical cases. These indicators would allow the estimation of the economic burden of surra in Tunisia and would help raise awareness among animal owners, veterinarians and animal health decision-makers. The epidemiological data would also allow for prioritisation of this disease among several other dromedary diseases, such as camelpox, mange, ringworm, gastrointestinal helminths, or would help to trigger integrated control of such diseases [10]*.* Like surra, some of these diseases are zoonotic [38], making prioritisation more complicated since human health could be included as a criterion.

### Control of surra in Tunisia

The use of a Progressive Control Pathway (PCP) approach, currently being promoted for Nagana/African animal trypanosomoses, could also enhance actions against surra by establishing clear, step-wise goals and milestones [10].

A programme mainstreaming the PCP approach does not need to aim for elimination of the infection, since this goal is probably unrealistic in many areas, at least in the foreseeable future. This is due to several factors, including the permeability of borders with neighbouring countries. This permeability is notably due to illegal trade of animals with neighbouring countries. For this reason, a transnational programme including all the North African countries should be established to target a significant reduction of surra infection prevalence at the regional level.

To guarantee the best use of resources allocated to control programmes, knowledge attitudes and practices (KAP) surveys should be performed to identify specific constrains facing the stakeholders. A KAP survey performed for other parasitic diseases in Tunisia showed unexpected data generation that could help improving the effectiveness of specific control programmes and shorten the time period needed for the elimination of different animal diseases [16, 19, 23]. If properly warned, the dromedary owners could be encouraged to present their animals during the early stages of the disease. This would avoid the increase in parasitaemia that enhances irreversible pathogenic effects and favours the spread of parasites through biting flies acting as mechanical vectors.

## Conclusion

In Tunisia, surra is enzootic in dromedaries in all the southern parts of the country, causing high production losses. Several aspects on diagnosis and control could be improved to decrease the impact of this parasite on animals. We conclude that surra research has a lot to learn from animal and human African trypanosomosis. In other words, several works and approaches used to study and control this *Trypanosoma* group disease could be adapted to and adopted for surra.
